# Perioperative myocardial injury is associated with increased postoperative non-cardiac complications in patients undergoing vascular surgery: a post hoc analysis of a randomised clinical pilot trial

**DOI:** 10.1186/s13741-023-00350-y

**Published:** 2023-11-13

**Authors:** A. Valadkhani, A. Gupta, M. Bell

**Affiliations:** 1 Perioperative Medicine and Intensive Care (PMI), Karolinska University Hospital, Stockholm, Sweden; 2 Department of Pharmacology and Physiology, Karolinska Institutet, Stockholm, Sweden

**Keywords:** Myocardial injury, Vascular surgery, Postoperative complications

## Abstract

**Background:**

Elevated cardiac biomarkers, such as high-sensitivity cardiac Troponin T and N-terminal pro-B-type natriuretic peptide improve the prediction of major adverse cardiac events. However, very few trials have investigated the association between perioperative cardiac injury and non-cardiac complications. The primary aim of this study was to determine the association between peri-operative myocardial injury and non-cardiac complications in patients undergoing vascular surgery. Additionally, the association between elevated pre-operative high-sensitivity cardiac Troponin T or N-terminal pro-B-type natriuretic peptide and non-cardiac complications was explored.

**Methods:**

This study is a post hoc analysis of a multicentre randomised controlled trial. Patients were recruited from three centres in Sweden between 2016 and 2019. Cardiac troponin level was measured pre-operatively and at 4, 24, and 48 h after the start of surgery in patients undergoing vascular surgery. N-terminal pro-B-type natriuretic peptide was measured pre-operatively. The primary outcome was a composite of major postoperative non-cardiac complications assessed at 30 days.

**Results:**

A total of 184 patients undergoing peripheral or aortic vascular surgery were included in this study. The primary endpoint occurred in 67 (36%) patients. Perioperative myocardial injury was significantly associated with non-cardiac complications, with an adjusted odds ratio (OR) of 2.71 (95% confidence interval 1.33–5.55, *P =* 0.01). Sensitivity and specificity were 0.40 and 0.81, respectively. No association was found between pre-operative hs-cTnT or NT-proBNP and non-cardiac complications.

**Conclusion:**

In this pilot study, we found that new peri-operative myocardial injury is associated with an increased risk of non-cardiac complications within 30 days after index surgery in patients undergoing vascular surgery. Pre-operative high-sensitivity cardiac Troponin T or N-terminal pro-B-type natriuretic peptide did not appear to predict non-cardiac complications. Larger studies are needed to confirm our findings.

**Trial registration:**

EudraCT database: 2016-001584-36

## Introduction

Pre-operative risk assessment tools are recommended to identify patients who are at high risk of peri-operative complications (Wanhainen et al. 2019; Kolh et al. 2016). Pre-operative risk assessment before non-cardiac surgery has recently been evaluated using biomarkers. Elevated pre-operative N-terminal pro-B type natriuretic peptide (NT-proBNP) and high-sensitivity cardiac Troponin T (hs-cTnT) have been associated with increased postoperative mortality (De Hert and Lurati Buse 2020; Puelacher et al. 2018). Peri-operative myocardial injury (PMI) is associated with increased mortality and adverse cardiovascular outcomes (Puelacher et al. 2018; Devereaux et al. 2017; Thygesen et al. 2018; Chew et al. 2023). Several studies have explored the association between PMI and postoperative cardiac complications and their use for perioperative risk assessment is now recommended in the current guidelines (Halvorsen et al. 2022).

Morbidity and mortality associated with postoperative major adverse cardiac events are similar to postoperative major adverse non-cardiac events (Beattie et al. 2018). However, comparatively few have studied the association between PMI and non-cardiac complications (NCC). One large cohort study showed an association between PMI and non-cardiovascular death (Devereaux et al. 2012). Noordzij et al. found an association between a 100 % postoperative increase in troponin and NCC (Noordzij et al. 2015). Patients undergoing vascular surgery have a higher risk of postoperative complications including NCC compared to other surgical cohorts, due to associated comorbidities (Healey et al. 2002). Furthermore, it has been shown that pneumonia and bleeding have the highest impact on clinical outcomes after vascular, abdominal or lower extremity surgery (Bennett et al. 2017). The primary aim of the present study was to determine the association between *peri-operative* myocardial injury and non-cardiac complications in patients undergoing vascular surgery. We also explored the association between *pre-operative* elevated cardiac biomarkers, hs-cTnT or NT-proBNP and NCC.

## Methods

This study is a *post hoc* analysis of data from our previously published study (Valadkhani et al. 2022), which was a prospective, multicentre, single-blinded, randomised clinical pilot trial. The study was approved by the Medical Drug Protection Agency in Uppsala, Sweden, (registration number: 5.1-2016-30176). Ethical approval for this study (registration number: 2014/310/1-3) was provided by the Swedish Ethical Review Authority, Uppsala, Sweden (Chairperson Per-Erik Nistér) on 27 August 2014. It was also registered in the EudraCT database: 2016-001584-36.

### Patients and study procedure

Patients > 60 years of age undergoing peripheral vascular or aortic surgery and without communication or cognitive difficulties were included in the study. The exclusion criteria were chronic obstructive pulmonary disease (COPD), other lung diseases that result in a patient requiring supplemental oxygen at rest, arterio-venous fistula surgery, carotid surgery or being enrolled in other clinical trials. Written informed consent was obtained from patients deemed eligible from three centres in Sweden: Karolinska University Hospital Stockholm, University Hospital, Linköping and Central Hospital, Karlstad. Recruitment started in November 2016 and was concluded in November 2019.

Randomisation was accomplished by inserting computer-generated random numbers in opaque and sealed envelopes, designated to each site. Patients were randomised in a 1:1 ratio to Group Normoxia (F_i_O_2_ = 0.21 and SpO_2_ target > 90%) and Group Hyperoxia (F_i_O_2_ = 0.50 and SpO_2_ target > 98%). Patients, surgeons assessing complications, and biochemical analysts were blinded to group allocation. Biomarkers including NT-proBNP and hs-cTnT were acquired before surgery (on the day of the index surgery) and 4, 24, and 48 h after the start of surgery. These biomarkers were measured using *Cobas Immunoanalyser, Roche Diagnostics, Rotkreutz, Switzerland*. Postoperative complications within 30 days were registered in medical records by surgeons and later validated by one of the authors.

### Outcome and definitions

The primary outcome was non-cardiac complications (NCC) occurring within 30 days after index surgery. A NCC was defined as one or more complications included in the clusters presented in Table 1.

**Table 1:** Postoperative non-cardiac complications clustered in different groups

Complication	Definition
*Neurological*	Stroke/transient ischaemic attack (TIA): diagnosis was based on radiological imaging of the brain and clinical examination. Postoperative delirium: diagnosis was based on the criteria in the fifth edition of the Diagnostic and Statistical Manual of Mental Disorders (DSM-5) (Boustani et al. 2014).
*Renal*	Acute kidney injury (AKI): an output of urine < 0.5 ml kg^−1^ h^−1^ for at least 6 h or a postoperative creatinine ≥ 1.5 higher than pre-operative baseline (Khwaja 2012).
*Bleeding or thrombo-embolic*	Major peri-operative bleeding: transfusion of over four units of red cell concentrates during 24 h. Transfusion due to intraoperative bleeding was not included. Deep vein thrombosis/pulmonary embolism: the diagnosis was based on clinical suspicion confirmed by ultrasound (duplex) or radiological examination. Severe coagulopathy: coagulopathy which requires pharmacological intervention (excluding pulmonary embolism or deep vein thrombosis).
*Infectious*	Surgical site infection (< 30 days): diagnosis was based on criteria established by the Centre for Disease Control and Prevention (CDC) (Mangram et al. 1999). Urinary tract infection: based on clinical presentation and urine cultures. Pneumonia: based on clinical presentation, radiological examination, and airway cultures. Other infections: not mentioned above and that required antibiotic treatment.
*Other*	Other complications: complications that were considered by the investigator as serious, delayed time to discharge from the hospital or resulted in readmission

### Non-cardiac complications (NCC)

#### Peri-operative myocardial injury (PMI)

PMI was defined as either acute PMI or acute chronic PMI, as defined below.

#### Acute PMI

Normal hs-cTnT values pre-operatively and postoperative increase by > 14 ng l^−1^ (Thygesen et al. 2018).

#### Chronic PMI

Pre-operative hs-cTnT > 14 ng l^−1^ and postoperative hs-cTnT < 14 ng l^−1^ (Puelacher et al. 2018).

#### Acute on chronic PMI

Pre-operative hs-cTnT > 14 ng l^−1^ and an increase in postoperative hs-cTnT by ≥ 14 ng l^−1^ from the pre-operative value (Puelacher et al. 2018).

### Statistics

Patients were grouped by whether they had a non-cardiac complication or not. Categorical values are presented as numbers and percentages. Continuous variables are presented as median and interquartile range (IQR). *P* values were calculated using Mann-Whitney *U* test and chi-squared test as appropriate. The association between pre-operative hs-cTnT and NT-proBNP NCC was assessed using generalised linear regression with binomial family. Since the outcome of this post hoc analysis was altered compared to the original study and did not include cardiac complications, we chose to use multiple regression to adjust for oxygen or air. Further adjustments were made using multiple regression to account for the difference in endovascular surgery and duration of operation between the groups with and without NCC. Generalised estimating equation (GEE) regression models were used to account for the statistical correlation of the repeated measurement of hs-cTnT when estimating the association between PMI and NCC, in patients where hs-cTnT was available. The GEE model was further adjusted for air or oxygen therapy, endovascular surgery and duration of operation. Based on the fitted model, sensitivity and specificity were estimated. A GEE model was used to account for which cluster of complications most strongly accounted for the association between PMI and NCC. Multiple regression was used to compare the association between PMI and NCC with the association between body mass index (BMI), duration of operation, American Society of Anaesthesiologists (ASA) score and intra-operative haemorrhage > 500 ml, as independent variables, and NCC. *P* values < 0.05 were considered statistically significant. All analyses were conducted using R version 4.1.2 (R Core Team (2021). R: A language and environment for statistical computing. R Foundation for Statistical Computing, Vienna, Austria)

## Results

In total, 184 patients were included in this study. The patient characteristics are presented in Table 2. There was a statistically significant difference between the groups with no NCC and NCC in patients undergoing endovascular surgery *vs*. non-endovascular surgery (39 % *vs*. 24 %, *P =* 0.049) and in the duration of operation (155 min *vs.* 230 min, *p* < 0.001), see Table 2.

**Table 2:** Patient characteristics

	No complications	Non-cardiac complications	*P* value
*n*	117	67	
Age (median [IQR]) years	74 [69, 78]	75 [70, 80]	0.576
Sex, *n* (%) male	86 (73)	48 (71)	0.919
BMI (median [IQR]) kg m^−2^	26 [24, 30]	25 [24, 29]	0.333
American Society of Anaesthesiologists classification			0.333
2	27 (23)	11 (16)	
3	88 (75)	53 (79)	
4	2 (1)	3 (4)	
Surgical procedure			
Open aortic surgery	13 (11)	11 (16)	0.423
Peripheral vascular surgery	58 (50)	40 (60)	0.241
Endovascular aortic surgery	46 (39)	16 (24)	0.049
Anaesthesia technique			
General anaesthesia	40 (34)	31 (46)	0.144
Regional anaesthesia	79 (67)	39 (58)	0.268
Intra-operative haemorrhage > 500 ml	24 (20)	21 (31)	0.143
Comorbidities			
Smoker	38 (32)	25 (37)	0.615
Hypertension	101 (86)	57 (85)	0.989
Diabetes, no insulin therapy	26 (22)	14 (20)	0.957
Diabetes, with insulin therapy	14 (12)	11 (16)	0.547
Chronic obstructive pulmonary disease	20 (17)	10 (14)	0.841
Heart failure	19 (16)	14 (20)	0.571
Ischemic heart disease	44 (37)	21 (31)	0.461
Stroke or TIA	10 (8)	10 (14)	0.284
Cancer	11 (9)	10 (14)	0.383
Chronic kidney disease	21 (18)	8 (11)	0.374
Biomarker, pre-operative			
Creatinine (median [IQR]) μmol l^-1^	85 [72, 101]	82 [68, 98]	0.298
NT-proBNP (median [IQR]) ng l^-1^	219 [97, 537]	231 [121, 539]	0.440
hs-cTnT (median [IQR]) ng l^-1^	13 [9, 18]	13 [10, 18]	0.584
Duration of surgery (median [IQR]) minutes	155 [118, 231]	230 [163, 299]	<0.001
Group hyperoxia^a^	61 (52)	29 (43)	0.316

All results are presented as *n* ( %), unless otherwise stated. *IQR* = interquartile range

*Abbreviations*: *NT-proBN P* N-terminal-pro-B-type natriuretic peptide, *hs-cTnT* High-sensitivity cardiac Troponin T

^a^Group hyperoxia (F_i_O_2_ > 0.5) or normoxia (F_i_O_2_ = 0.21)

In two patients, no pre-operative hs-cTnT was obtained while one had no postoperative hs-cTnT. A statistically significant association was found between *peri-operative* myocardial injury (PMI) and NCC at 30 days after index surgery with an adjusted OR of 2.71 (95 % CI 1.33–5.52, *P =* 0.01). Sensitivity and specificity were 0.32 and 0.87 respectively. The incidence of complications in each cluster, other than death, is shown in Table 3. One patient in the NCC group died within 30 days after index surgery. When comparing different clusters, PMI was most strongly associated with renal complications (*P =* 0.03).

**Table 3:** Association between PMI and each cluster of non-cardiac complication

Non-cardiac complications	Number (%) of complication	Adjusted OR	*P* value
*Neurological*	2 (1)	2.72 (0.10–74.40)	0.55
*Renal*	10 (5)	4.33 (1.13–16.33)	0.03
*Bleeding or thrombo-embolic*	10 (5)	0.73 (0.15–3.48)	0.69
*Infectious*	29 (16)	1.47 (0.60–3.63)	0.40
*Other*	36 (20)	2.00 (0.92–4.36)	0.08

Generalised estimating equation (GEE) regression models were used to adjust for repeated measurement of troponin, intervention (hyperoxia vs. normoxia), endovascular surgery and duration of surgery. Please see Table 1 for the definition of each cluster

*PMI* = peri-operative myocardial injury

Pre-operative hs-cTnT and NT-proBNP were missing in two and three patients respectively. These patients were excluded from the regression models. The association between clinical characteristics and non-cardiac complications are shown in Table 4. No association was found between pre-operative hs-cTnT or NT-proBNP and NCC. A significant association was found between duration of surgery > 120 min and NCC, with an OR of 3.33 (95 % CI 1.17–9.47, *P =* 0.02).

**Table 4:** Association between clinical characteristics and non-cardiac complication

Variable	Unadjusted OR	*P* value	Adjusted OR	*P* value
Pre-operative hs cTnT	1.08 (0.81–1.45)	0.60	1.06 (0.77–1.44)	0.73
Pre-operative NT-proBNP	1.36 (0.93–1.97)	0.11	1.36 (0.90–2.04)	0.15
PMI	2.85 (1.44–5.65)	< 0.01	2.71 (1.33–5.52)	0.01
ASA classification	1.62 (0.81–3.26)	0.17	1.83 (0.88–3.81)	0.10
BMI > 30	0.77 (0.37–1.60)	0.48	0.81 (0.37–1.78)	0.60
Intra-operative bleeding > 500 ml	1.77 (0.89–3.51)	0.10	1.26 (0.61–2.61)	0.53
Duration of surgery > 120 min	3.69 (1.45–9.40)	0.01	3.33 (1.17–9.47)	0.02

*PMI*: generalised estimating equation (GEE) regression models were used to adjust for repeated measurement of troponin, intervention (hyperoxia vs. normoxia), endovascular surgery and duration of surgery. Other variables: logistic regression was used for unadjusted analysis and multiple regression analysis was used to adjust for intervention in the HYPEROXIA-trial, endovascular surgery, and duration of operation *Abbreviations*: *PMI* = peri-operative myocardial injury, *ASA* = American Society of Anaesthesiologists

## Discussion

We found an association between *peri-operative* myocardial injury (PMI) and the subsequent occurrence of a non-cardiac complication (NCC) in patients undergoing vascular surgery. However, we found no association between increased *pre-operative* hs-cTnT or NT-proBNP and NCC.

The definition of PMI varies depending on the thresholds used (Puelacher et al. 2018; Devereaux et al. 2017). It is important to distinguish between patients with *chronic PMI*. and those with *acute on chronic PMI*. This distinction is important since patients with an *acute on chronic PMI* have a higher mortality compared to those with a *chronic PMI* (Puelacher et al. 2018). Furthermore, this distinction was important in our patients undergoing vascular surgery since up to 40% had an elevated pre-operative hs-cTnT > 14 ng l^−1^(Valadkhani et al. 2022).

Compared to trials that have studied the association between PMI and cardiac complications there is a scarcity of trials that have addressed the association between PMI and NCC (Chew et al. 2023). Noordzij et al. studied patients undergoing abdominal surgery and found that an increase in hs-cTnT by 100% between pre-and postoperative values was associated with NCC(11). However, they could not demonstrate an association between elevated pre-operative hs-cTnT and NCC, which was similar to our finding. The sensitivity and specificity were also similar to our results, albeit in patients undergoing abdominal surgery (Noordzij et al. 2015). In a recent study, the authors found an association between PMI and NCC in patients undergoing non-cardiac surgery. However, they did not distinguish between chronic PMI and acute chronic PMI, which is problematic (Ackland et al. 2020). It is important to stress that patients with PMI who have an NCC postoperatively also have higher mortality compared with those who have NCC alone (Beattie et al. 2018; Ackland et al. 2020). Therefore, the detection of PMI may contribute to the prognostication of postoperative mortality.

Our findings also show that PMI was most strongly associated with the cluster of renal complications, which was similar to the findings of Ackland et al. (2020). Previous studies indicate that patients with acute kidney injury can have an acute rise in troponin that may be mistaken for PMI (Banerjee et al. 2019). However, hypotension and sepsis may also cause acute kidney injury so it is possible to have a simultaneous acute kidney injury and PMI (Halvorsen et al. 2022; Banerjee et al. 2019). Despite statistical significance, the absolute number of patients who had renal complications in our study was small and therefore this finding should be interpreted cautiously.

There is no simple physiological explanation for the association between PMI and NCC since PMI is multifactorial (Halvorsen et al. 2022). If PMI occurs following a cardiac event (e.g., arrhythmia, demand-perfusion mismatch), it is possible that perfusion to other organs might be affected, which may in turn lead to NCC. In contrast, non-cardiac causes of PMI such as sepsis or pulmonary embolism might lead to an NCC. Therefore, several distinct pathways may explain how PMI leads to NCC. The temporal relationship between peri-operative PMI and NCC could suggest that PMI increases the risk of NCC. However, it is also possible that both PMI and NCC occur independently as a consequence of an intra-operative event such as hypotension (Sessler et al. 2019).

Recent guidelines have concluded that elevated pre-operative NT-proBNP is associated with significantly increased postoperative mortality (Halvorsen et al. 2022). Furthermore, it is also associated with cardiac complications (Karthikeyan et al. 2009). However, the association between NT-proBNP and NCC remains unexplored, to the best of our knowledge. In addition to the above cardiac markers, other factors such as American Society of Anaesthesiology Physical status, intra-operative blood loss and duration of surgery have been shown to be associated with postoperative complications (Glance et al. 2011; Cheng et al. 2018; Hopkins et al. 2016). In our study, the odds ratio for PMI was greater than all the other factors known to be associated with postoperative complications, except the duration of surgery.

This study has several strengths. It is based on a prospective, multicentre randomised controlled trial with blinded outcomes registered by surgeons. Moreover, we have biomarker data collected both before and after surgery whereby acute, chronic and acute on chronic PMI could be clearly differentiated. This study also focuses on a cohort of patients undergoing vascular surgery who are at high risk of developing postoperative complications (Healey et al. 2002). Finally, the definition of PMI used in this study is the same as recommended in current guidelines and differentiates between acute and acute on chronic PMI. There are some limitations to our conclusions, based on sample size and methodological issues. It is a relatively small study and is based on secondary analysis of data, which could introduce unknown bias. Additionally, we did not attain 48-h hs-cTnT in all patients since some were discharged earlier. A further weakness of this study is the absence of high-resolution intra-operative hemodynamic data that may give a better insight into the aetiology of PMI.

In conclusion, we found an association between PMI and non-cardiac complications within 30 days after index surgery in patients undergoing vascular surgery. This strengthens the importance of troponin measurement in the pre- and postoperative period since it can be used to assess the risk of developing non-cardiac complications after vascular surgery. The physiological causal relationship between PMI and NCC remains unclear and is likely to be multifactorial.

## Data Availability

The datasets generated and/or analysed during the current study are not publicly available due to the risk of compromising patient privacy but are available from the corresponding author on reasonable request.

## Abbreviations

POSSUM: Portsmouth-Physiological and Operative Severity Score for the enUmeration of Mortality and morbidity
RCRI: Revised Cardiac Risk Index
MACE: Major adverse cardiac events
NT-proBNP: N-terminal pro-B type natriuretic peptide
PMI: Peri-operative myocardial injury
NCC: Non-cardiac complications
hs-cTnT: High-sensitivity cardiac Troponin T
COPD: Chronic obstructive pulmonary disease
IQR: Interquartile range
GEE: Generalised estimating equation
BMI: Body mass index
ASA: American Society of Anaesthesiologists
